# Evaluating a research training programme for frontline health workers in conflict-affected and fragile settings in the middle east

**DOI:** 10.1186/s12909-023-04176-6

**Published:** 2023-04-13

**Authors:** Hady Naal, Tracy Daou, Dayana Brome, Rania Mansour, Ghassan Abu Sittah, Christos Giannou, Enrique Steiger, Shadi Saleh

**Affiliations:** 1 Global Health Institute at the American University of Beirut, Beirut, Lebanon; 2 St George’s Hospital Medical School, St George’s University of London, London, UK; 3 Queen Mary University of London, London, UK; 4 Swiss Cross Foundation, Zürich, Switzerland; 5 Faculty of Health Sciences at the American University of Beirut, Beirut, Lebanon

**Keywords:** Conflict, Health research, Global health, Capacity building, Antimicrobial resistance, LMIC, Middle east

## Abstract

**Background:**

Health Research Capacity Building (HRCB) is key to improving research production among health workers in LMICs to inform related policies and reduce health disparities in conflict settings. However, few HRCB programmes are available in the MENA region, and few evaluations of HRCB globally are reported in the literature.

**Methods:**

Through a qualitative longitudinal design, we evaluated the first implementation of the Center for Research and Education in the Ecology of War (CREEW) fellowship. Semi-structured interviews were conducted with fellows (n = 5) throughout the programme at key phases during their completion of courses and at each research phase. Additional data was collected from supervisors and peers of fellows at their organizations. Data were analysed using qualitative content analysis and presented under pre-identified themes.

**Results:**

Despite the success of most fellows in learning on how to conduct research on AMR in conflict settings and completing the fellowship by producing research outputs, important challenges were identified. Results are categorized under predefined categories of (1) course delivery, (2) proposal development, (3) IRB application, (4) data collection, (5) data analysis, (6) manuscript write-up, (7) long-term effects, and (8) mentorship and networking.

**Conclusion:**

The CREEW model, based on this evaluation, shows potential to be replicable and scalable to other contexts and other health-related topics. Detailed discussion and analysis are presented in the manuscript and synthesized recommendations are highlighted for future programmes to consider during the design, implementation, and evaluation of such programmes.

**Supplementary Information:**

The online version contains supplementary material available at 10.1186/s12909-023-04176-6.

## Introduction

Over the past few decades, the Middle East and North Africa (MENA) region has been inflicted with multiple armed conflicts and civil wars resulting in devastating humanitarian crises and overwhelming waves of forced migration. As a result, six MENA countries (Iraq, Libya, Lebanon, Palestine, Syria, Yemen) have been included in the World Bank’s list of fragile and conflict affected situations (FCAS), which includes countries directly or indirectly affected by violent conflicts as well as countries experiencing high levels of institutional and social fragility [1]. Not only have the widespread humanitarian crises throughout the region become more frequent, but they have also become protracted, complex, and costly, resulting in negative consequences on the health of civilians and posing a large burden on healthcare systems [2–4].

The population health outcomes of FCAS in the MENA region are multifactorial. Initially, acute health problems and demanding health needs arise due to trauma, injuries, spread of infectious disease, and compromised access to health services due to fragmented health systems. Gradually, as healthcare systems are overwhelmed, long term and multifaceted problems are likely to emerge which exacerbate population health outcomes; these include loss of livelihood, massive displacement, breakdown of social services, social disruption, and economic decline, which altogether hinder the re-implementation of basic services. Health problems are thus exacerbated not only by the emergence of health conditions that require specialty care, but also by systemic factors that overwhelm the health system and impact health protocol and policy development as well as long term health planning. For instance, more than 800 incidences of violence against healthcare were reported in FCAS in 2020, with more than 180 healthcare workers killed [5]. Such security threats, among others, have led to the massive migration of qualified healthcare workers from their conflict-affected countries [6, 7]. In Syria for example, 70% of healthcare workers have fled the country, with reports indicating that security concerns were the primary driving force [8, 9]. As a result of this brain drain, civilians are frequently left without adequate care, which contributes to the rise in mortality and morbidity among conflict-affected populations [10–13]. For example, the rates of maternal and child mortality are higher in FCAS, with children in FCAS being twice as likely to die before the age of 5 years old compared to children in stable low- and middle-income countries (LMIC) [14, 15]. Similarly, both the acute and long-term impact of protracted conflict has made infectious disease management especially difficult. This is exemplified by the recurrent cholera outbreaks in Yemen, whereby approximately half of the population lacks access to appropriate sanitation facilities or clean drinking water [16] and where 18 million people are in urgent need of sanitation and hygiene assistance [17].

The unique health challenges faced by FCAS require the development and implementation of evidence-based health policies and interventions to improve the provision of health services and overall health of civilians. Health research in FCAS is especially pivotal in prioritizing health concerns, determining the efficacy of health interventions, and identifying when and how to best deliver care in crisis settings [2, 18, 19]. Nevertheless, there are three significant challenges to conducting health research in FCAS. The first challenge relates to the research context: humanitarian organizations and healthcare systems in FCAS are preoccupied with priorities relating to defence, infrastructure, and survival needs of their populations, resulting in an underdeveloped research culture [20]. Similarly, the public health information systems from which data can be extracted may be disrupted and/or politically biased, which affects the pooling, standardization, and management of data that eventually informs national health estimates and outcomes [2, 21, 22]. In the MENA region, government approval is sometimes required before population data can be collected and disseminated as such data may be considered a security threat by armed groups [23]. Indeed, there are laws in place penalizing researchers and reporters who criticize government actions or reveal such sensitive information, as has happened during the COVID-19 pandemic [20, 24]. The second challenge pertains to the research personnel, who may not possess the skills, expertise, or logistics to conduct high quality research while working in unstable settings due to security threats, politicization of data, underfunding, and a damaged healthcare system [2]. Coupled with the brain drain experienced by FCAS, it is unsurprising that research capacity in the MENA region is lacking [25], as evidenced by the few health research capacity building initiatives in the region [18]. The third challenge concerns the quality of research evidence disseminated. The inherent context of conflict settings poses a challenge to the type and quality of study design researchers can utilize to collect data. Amid a crisis or conflict, baseline data may be unavailable, control groups may be impractical, coordinating with local and international actors may be difficult, and obtaining informed consent from vulnerable or distressed participants may be unethical [2, 19].

A scoping review detailing the characteristics of health research capacity building programmes in the region affirms significant challenges in pursuing research in FCAS, with the most common obstacles being the lack of research culture, shortages in logistics, limited evaluations of health research programmes, and interpersonal difficulties [18]. Such challenges have collectively limited the opportunity to collect and analyse health research and subsequently implement evidence-based health interventions and policies. As such, humanitarian practice is often dependent on either anecdotal experience or research from stable settings [26], neither of which can be translated adequately to respond to the unique health challenges faced by FCAS. This is represented by the scarce evidence behind public health interventions in the region. A systematic review exploring the public health interventions in humanitarian settings documented weak quality and an overwhelmingly limited quantity of health intervention research within humanitarian crises [27]. Another systematic review exploring the global health capacity building initiatives in LMICs of the MENA region found gaps between the topics of initiatives and the region’s health needs, with only a few initiatives addressing conflict and emergency topics despite the overwhelming burden of conflict in the region [28]. Additionally, a review examining methods to evaluate such global health capacity building initiatives in LMICs concluded that there is a lack of standardized tools through which initiatives can be assessed, particularly for innovative capacity building programmes that go beyond in-person modalities; as a result, it is difficult to determine and compare the efficacy and efficiency of global health initiatives on improving health outcomes [29].

Given these challenges, it is crucial to strengthen the capacity of researchers in FCAS. The Inter-Agency Standing Committee (IASC) Reference Group for Mental Health and Psychosocial Support in Emergency Settings has set forth recommendations for conducting research in crisis settings. Particularly, IASC reported that research questions should: respond to a recognised gap or need; ensure fair and direct benefits to participants with minimized risks; and include planned dissemination to stakeholder. [2, 30]. Strategies for strengthening health research in FCAS have also been proposed by local researchers [2, 18, 27, 28] and include building local research capacity, strengthening regional collaborations, engaging with local communities, and relying on local leadership.

The Center for Research and Education in the Ecology of War (CREEW) based at the Global Health Institute (GHI) of the American University of Beirut (AUB) incepted a mentorship-based Fellowship as one of its programmes to respond to the aforementioned gaps based on the above recommendations. This was partly motivated by the lack of involvement of LMIC researchers addressing health challenges relevant to FCAS, as evidenced by a study in Lancet Global Health, which revealed that despite the fact that 92% of articles address interventions in LMICs, only 35% of authors are from LMICs [31]. As such, the CREEW Fellowship aims to foster local research production by equipping frontline health practitioners working in conflict settings with skills necessary to conduct research into the relationship between health and war. Humanitarian settings provide an opportunity to uniquely examine the ecology of war, which is a result of protracted conflict and involves a unique interaction between social, ecological, environmental, or economic factors and multiple domains including physical and mental health, education, livelihood, among others [3, 22]. As such, the research outputs of the Fellowship programme aim to inform the policy and practice of humanitarian and public health work in FCAS. Accordingly, this study reports findings of the short and long-term effectiveness and impact of the first implementation of the CREEW Fellowship.

## Methods

### Participants

Following an extensive application process that involved meeting the eligibility criteria, interviews with select candidates regarding their feasibility of their research topic, and a deliberation process by the admission committee that included a rubric to guide ratings of candidates, seven individuals were accepted into the programmeme. Only five were able to make it to Beirut from Iraq, Palestine, Sudan, Syria, and Yemen and were part of this cohort. Their details are outlined in Table 1. A scholarship that covered the costs of on campus courses, mentorship fees, lodging, travel, transportation, and research costs was offered to these five fellows.

**Table 1:** Overview of Participant Details

Nationality	Background	Site of mentored research	Study
Iraqi	Water & Sanitation Engineer	Iraq	A cross-sectional study assessing water samples for the presence of AMR bacteria in a health care setting in Baghdad
Palestinian	Public Health Specialist	Palestine	A cross- sectional study on antimicrobial resistant bacteria in water samples in a health care setting in the Gaza Strip
Yemeni	Medical Doctor	Yemen	A quasi-experimental intervention study on the impact of antimicrobial stewardship in a conflict setting
Sudanese	Medical Doctor	Sudan	A literature review on pharmaceutical policy and practices in Sudan and their relation to antimicrobial resistance
Syrian	Public Health Specialist	Turkey	A qualitative study on experiences of war-wounded Syrians whose injuries are affected by antimicrobial resistance

### Study design

The present study followed a qualitative longitudinal design with the aim of exploring in-depth the experiences of fellows throughout the course of the programme, while utilizing the Kirkpatrick model of programme evaluation (which conceptualizes evaluation of such programmes at the levels of reaction, learning, behavior, and results) as a guiding model to inform the current approach. Since very few initiatives have been conducted to improve the research skills of health workers in conflict settings, and because even fewer evaluations are available in peer-reviewed journals, it was very important for this present work to follow a longitudinal design [32].

The five fellows were contacted throughout the programme including during: (1) the inception phase of their research projects where they designed their data collection tools, (2) their application to the IRB, (3) their data collection phase, (4) their data management and data analysis phases, and (5) the write-up and dissemination phase. Each data collection point symbolized a key milestone in their research journey. Data was collected from the fellows via semi-structured interviews. These interviews inquired about their overall experience with the given research phase and focused on the main successes and challenges faced therein. The same data collection tool was used during each of the phases mentioned above.

Moreover, given that each fellow followed a different timeline to complete their research project due to contextual challenges or differing methodologies, time-points for collecting data from each fellow differed; as such, the guiding cue for data collection was completion of the respective research milestones. Additionally, an exit interview was conducted following graduation from the fellowship. It aimed to explore fellows’ experiences with the overall programme with a focus on the learning modality as well as knowledge and practice gains from the fellowship. At the end of fellowship, the team also contacted one colleague from each of the fellows’ organizations in order to inquire about potential changes in practice and/or knowledge demonstrated by the fellow during and/or after completion of the fellowship.

### Instruments

A qualitative was used to collect short-term and long-term individual-level and organizational-level data. The researchers who collected the data and carried out the evaluation interviews were separate from the staff who mentored, coordinated, or delivered the training.

After obtaining consent from learners and their colleagues, the research team at GHI contacted them through email correspondence to invite them to participate in the study. As part of this evaluation, data was collected from all five participants that were part of this cohort. Prompts were collected at the end of every phase of the research project through semi-structured interviews. After production of the research output after completion of the fellowship, fellows were invited for an exit semi-structured interview, and they were asked to nominate one colleague from their organizations to complete a survey regarding their performance at work. These organizational surveys were collected 7 months after completing the diploma through the contact information that was provided by learners.

#### Prompts at key research phases

During the mentorship phase, five prompts were collected from each learner at the end of the following research stages: (1) proposal development, (2) IRB preparations, (3) data collection, (4) data analysis, and (5) manuscript write-up. Prompts aimed to collect data on the successes, challenges, and barriers faced by fellows while completing each phase of their research. Invitation emails were sent to all five participants, and interviews were conducted over Zoom platform and recorded following the participants’ consent. Interviews were carried out in English, Arabic, or a mixture of both, depending on the preference of each fellow.

#### Exit semi-structured interviews

Following production of research output, semi-structured interviews were carried out within one month in order to assess the long-term impact of the CREEW fellowship on participants (See Table 2). The interviews aimed to collect data on learners’ knowledge, practices, and experiences in applying research methods in conflict settings, and their ability to disseminate their research. Interview invitation emails were sent out to all 5 participants. The interviews were administered in English, Arabic, or a mixture of both depending on the preferences of the fellows. All interviews were conducted through zoom and recorded after obtaining participant’s consent.

**Table 2:** Semi-structured interview guide

1. Describe your learning experiences during your participation in the training.
2. How did the online learning modality influence your learning process?
3. Describe your knowledge in research and education in the ecology of war after your participation in the training.
4. Describe your practices in research and education in the ecology of war after your participation in the training.
5. How did the training impact your capability to learn new skills?
6. What are the strengths and weaknesses of the training programme?
7. Since completing the training, have you been able to apply the research methods addressed in your practice?
8. To what extent were the courses contextualized/relevant to conflict settings?
9. Since completing the training, have you disseminated your research in locally or internationally through conferences or journal publications

#### Organizational-level surveys

Organizational surveys were sent to fellows’ colleagues, who were deemed able to comment on the fellows’ performance within the organization. The aim of these surveys was to assess the transfer of knowledge and skills in the fellow’s work environment. The survey included 5 questions rated on a Likert-scale, and 3 open-ended questions. For the purposes of this study, we only reported the open-ended questions given the small sample size. The open-ended questions focused on (1) fellows’ performance within the organization, (2) the strengths and weaknesses of the training, and (3) any perceived barriers that might have limited the application of the learners’ acquired skills into the organization following their participation in the training.

### Informed consent and ethical considerations

All participants provided written consent before taking part in research activities. This study was approved by the Institutional Review Board (IRB) at the American University of Beirut.

### Analysis

Analysis of qualitative data was conducted through qualitative content analysis technique following a deductive approach [33, 34]. All data was transcribed verbatim into its original language by the research team. At first, open coding was independently completed by two researchers. Next, codes were grouped into broader categories depending on similarities and differences. Finally, these were reported under predefined themes. Two researchers were responsible for the analysis and revision of coded responses; they met regularly to discuss the outcome of these analyses.

## Results

In view of the present study’s longitudinal design, results are presented under predefined categories according to their sequential order throughout the implementation of the programme. Data from semi-structured interviews and prompts was triangulated with data obtained from organizational surveys. Each category describes the successes and challenges encountered in its respective phase and includes (1) course delivery, (2) proposal development, (3) IRB application, (4) data collection, (5) data analysis, (6) manuscript write-up, (7) long-term effects, and (8) mentorship and networking.

### Course delivery

All fellows first enrolled in online courses from their home countries and then attended in-person courses in Lebanon during the initial phases of the programme. These courses allowed the fellows to prepare and develop their research proposals and ultimately launch their research field work. In general, fellows reported that attending the courses was beneficial for them, mainly due to their multidisciplinary nature which equipped them with the necessary foundational knowledge needed to conduct research on AMR in conflict settings. Responses from organizational surveys also showed that learners’ colleagues believed the educational material and references provided to be useful. Indeed, fellows expressed much satisfaction with the courses because they combined theoretical and practical aspects of research, were clear and easy to understand, and provided credible information by experts in the field.

*P.1.Great experience when it comes to courses which were very hmm useful and informative in terms of their scientific background, research background, covering the scientific background to be hmmm as the courses were related to AMR, antimicrobial resistance in conflict settings in addition to research courses that cover qualitative and quantitative research, so it covers both the research and scientific topics that helped to help the researcher to move to go ahead with the research in conflict setting, and it was in addition it was specific or it applies the lenses related to conflict setting, so when it comes to ethical consideration for research, they provided the examples in conflict settings, so it was very helpful, and informative.*

That said, fellows also commented that the time allocated for the courses was unevenly distributed; some material was perceived to be basic with more than enough allocated course time, whereas other courses such as Advanced Statistical Analysis was more important yet not given enough time.

Mixed findings were reported regarding the learning modality through which courses were delivered. Online learning was described as a novel experience for all fellows, which was generally perceived as feasible from a technical standpoint, and useful for fellows to store and revert back to course material multiple times should the need arise to better understand them. Importantly, some in-person courses had to be re-delivered online when COVID-19 impacted Lebanon; as such, fellows mentioned that online learning was a key contingency measure for them to be able to resume their courses without significant interruptions.

*P.2. The online [modality] had an important benefit which is the ability to record and you can listen again and can heard what have been discussed later and you repeat 1, 2, 3 times to understand, this is also a good advantage for the online [modality]*.

Despite this advantage, fellows largely preferred in-person learning because they perceived it to be more engaging and interactive, which helped them better understand the material. Additionally, some participants mentioned that online learning was difficult because it required greater organization and discipline, particularly in view of their limited experience with such modalities.

*P.3. The face to face [modality] brought us into the very contact with excellent staff, very qualified, and very confident. I felt I could ask any question comfortably, and so this was like a space that I felt was a very positive learning experience and a well-suited space.*

### Proposal development

Directly after successful completion of the courses and based on shared research interest, fellows were matched with mentors who are experts in their fields. They were expected to negotiate their research topic, to develop a proposal, and to defend it in front of the scientific advisory committee. Although the majority of this phase was originally planned to happen in-person at AUB, including the proposal defence, the COVID-19 outbreak resulted in evacuating fellows to their respective countries and resuming the work remotely.

Overall, fellows mentioned that this phase allowed them to acquire essential knowledge both in terms of the literature on AMR, but also more importantly in terms of understanding how to structure research proposals, the different procedures involved in producing research, how to formulate research objectives and research questions, and how to choose appropriate research designs. Additionally, having the opportunity to present and defend their proposals to a scientific committee was perceived to be especially useful because of the feedback they received, which allowed them to perform major revisions and solidify their proposals.

*P.5. If you had asked me what the research steps are before coming to Beirut, I would not have known. I didn’t know that there are different types of research like experimental, quasi-experimental, I didn’t know anything before, and I have no idea. I was surprised to see myself picking up on all this knowledge in a small period of time.*

As for the challenges encountered in this phase, they were associated with planning for research in contexts of conflict, and with the ability to apply material learned to develop a research proposal. The first included challenges such as difficulty finding appropriate and relevant literature on AMR in the region, the need to change and adopt a methodological approach that is feasible in the given context of conflict especially following the outbreak of COVID-19, and the limited ability to collaborate with ministries of health to retrieve archival data, and local actors to receive other forms of local support for data collection.

*P.3. The first challenge had to do with the current context, where we are trying to do this research. The context being the Covid-19 measures that are taken by all the governments and my workplace for example different hospitals which reduced basically my ability to interact with them with partners and to track with my context, and this of course created the issues of logistics when it comes to processing the specimens or to accessing the facilities or who wants to do the research, so this is one issue. The second also, it’s also related to the Covid-19 measure and it is also related to the ongoing let’s say the geopolitical situation of today which is accessing the ministry of health and trying to get the information from it.*

Regarding the second category of challenges, some differences were observed among fellows. For example, while some mentioned that they felt ready to apply the knowledge gained on AMR and research methods to develop a research proposal, others reported uncertainty on how to integrate their theoretical understanding into a tangible research proposal. Still others mentioned that they believed their experience was not sufficient to design, implement, and produce a research output from their projects. Another mentioned that their lack of experience affected their ability to properly plan for and communicate the needed lab equipment and subsequently manage their data and perform the required analyses.

*P.2. Of course, given the COVID-19 pandemic, country unrest, and restricted mobility which impacted my work on the proposal, I felt that I didn’t have the full potential and the required knowledge to conduct a proposal at this level.*

These challenges were a priori expected to happen, and this is why fellows were paired with mentors to provide guidance throughout every step of the programme. Indeed, mentors were reported to play an important role in the fellowship, as testified by one fellow who faced critical challenges working on a medical topic that did not align well with his background. The fellow reported having to shift multiple times his research project and his proposed methodology because of the latter in addition to the obstacles imposed by the context where he was operating.

***P.4.*** *he [the mentor] showed interest and support and transfer all his knowledge and supporting to me and he was changing the subject of the research and I was accepting this because I have difficulty because I have the feeling that we will achieve something, but also for him he was surprised and shocked by the challenge that we faced, but from his side he was available, he was doing the correction, he was always advice to change the article the way of express by his better than me language, his language better than me and he have skill by reporting, he was improve what I produce, but at the end we could not succeed.*

### IRB application

This phase was generally perceived to be stressful for fellows primarily because their projects depended on securing ethics approval and they had to be ready to collect data in a relatively short period of time. In this regard, fellows expressed concerns about their projects not being approved, not being able to adjust their projects based on IRB’s comments, and not being able to obtain the required permissions for data collection needed by IRB from institutions in their home countries such as hospitals and local NGOs. In addition to these issues, fellows had to consider that they were conducting research in conflict settings during a global pandemic, in countries outside Lebanon, and among sensitive populations, all of which further complicated the process.

*P.4. To be honest, it was a challenge at the start to find a university that would offer ethical approval for our research proposal in the midst of Syria’s instability and conflicting context.*

Despite the presence of major concerns relating to the uncertainty of their local contexts, time constraints imposed by long IRB revision periods, and having to adjust their methodologies to account for COVID-19 restrictions, substantial positive outcomes were reported. During this phase, almost all fellows experienced for the first time the process of applying for ethics clearance, which added significant value to their learning experience in research. This included knowing how and where to apply for IRB approval, communicating with IRB personnel and responding to feedback, and better understanding the role of ethics in conducting research, especially among vulnerable and conflict-affected settings/populations.

*P.4. Looking over a handful of IRB application forms was an advantage that I was introduced to. This was particularly useful in terms of learning what are the required documents to include, such as a consent form and what information should be included in this form because, at the end of the day, there may be a harm imposed on the participants, so it is critical to consider the impact that your study will have on the community, whether positively or negatively. The consent allows you to be ethical in your study and considerate of the participants. So, this has a big impact on how you structure your study, what questions you can ask and what information you can get from the interviews. You should be considerate of how your research project will affect your participants.*

Overall, fellows reported adequate support from mentors in order to get through this process; however, many encountered significant delays in their research projects primarily because of this time-consuming process. This was one of the reasons why some fellows could not finish their projects on time and required extensions.

### Data collection

Multiple successes related to data collection were highlighted by fellows. For instance, one described how he was able to successfully reach the target sample size of participants in his study. He also noted that he gained skills in selecting his sample of participants based on the required characteristics with the capability to diversify it and ensure equal gender representation, allowing him to then stratify data by gender in a context where data from women is underrepresented in research. The fellow further indicated that he gained knowledge in conducting and managing interviews with increased understanding of how to steer interviews to obtain the necessary information. He also mentioned learning how to transcribe interviews despite time restraints.

*P.4. As I started to conduct more and more interviews, I began to experience an improvement in my ability to ask suitable questions to bypass any unnecessary answers from the participants. I attained experience in how to conduct and manage interviews…. The most significant success that I believe to have achieved is that after initially planning to recruit 10–15 participants in order to reach data saturation, I was able to successfully recruit and interview 14 participants with the required characteristics. I was able to accomplish this specifically during times of country unrest and instability.*

During this phase, fellows mentioned experiencing various challenges during recruitment, sampling, and conducting interviews. One fellow highlighted learning how to build working relationships with the lab experts during data collection when needing to secure materials for lab sample testing. By building these relationships, the fellow was able to obtain a second sample that was more representative than the one they initially utilized. Another fellow noted challenges in effectively testing for samples in hospitals due to unreliable test results obtained from hospital labs that showed very little expertise and cooperation; this also affected the communication and relationship between the lab members and the mentors.

One fellow highlighted having trouble in recruiting participants who met his inclusion criteria given that their contact information was missing from the hospitals, forcing him to travel across town to access the participants’ contact information and execute proper recruitment. Additionally, he indicated being forced to engage in further rounds of recruitment due to a high dropout rate among participants who initially provided their consent to participate. Additional challenges encountered during the interviews involved the need to conduct interviews for an extended period of time in order to reach saturation and to ensure appropriate quality of data from participants’ narratives was obtained. Importantly, internet connectivity issues impeded the scheduling and conducting of online interviews. Finally, the fellow also reported experiencing emotional distress when interviewing participants given that their stories revolved around living in war and conflict settings.

Almost all fellows reported that contextual challenges relating to COVID-19 restrictions, along with civil unrest within their countries, contributed to limiting the fellows’ mobility which was needed for data collection. One of them reported facing difficulties in collecting and storing data given the country’s political unrest and its impact on the health system. Others indicated that COVID-19 restrictions posed difficulty on their ability to visit hospitals to collect lab samples, or their ability to cross country borders to visit hospitals where they can collect samples.

*P.1. I felt like something was wrong. We discovered that the collected sample did not contain phyto chlorophyll or enterococcus, so we had to change it and perform another round of sampling; we also doubted that there was a mistake in the testing technique or a problem in how the samples were stored. So, sample testing and quality assurance had certain difficulties, which I had to overcome by doing a re-sampling.*

*P.4. To be honest, we were in a conflicted country that was constantly exposed to breakdowns, notably in its healthcare system and when dealing with data management and storage. So we faced difficulty in recruiting the target sample that had to be interviewed, especially that the participants’ data found in the hospitals was highly disorganized. At the same time, because our target sample was limited to individuals with antimicrobial resistance, it was difficult to trace who had antimicrobial resistance and who did not based on the available disorganized data.*

### Data analysis

Fellows also provided feedback on the data analysis phase, perceiving it to be successful overall. Fellows particularly mentioned refining their skills in quantitative data analysis, such as via using statistical analysis software, as well as improving their skills in conducting, transcribing, and thematically coding qualitative data.

*P.5. The most important achievement was that I was able to learn something new, such as how to use SPSS and how to deal with Excel sheets and perform certain functions that I had never done before. I gained skills in conducting analyses and reading bar and graph analysis outputs, which I consider to be personal achievements.*

*P.4. What I accomplished during this analysis phase is the ability to extract codes, themes, and categories from all the 14 interviews that I conducted. So what I accomplished is the formulation of a large document including 7,000 words incorporating the most important codes related to the infected participants in addition to the themes and categories that facilitated my upcoming tasks.*

One learner mentioned improving her capability to prepare data for analysis by categorizing the data into subfolders and inserting them into multiple SPSS sheets, which facilitated the production of significant and interpretable results. The fellow also expressed a newfound ability to interpret and draw conclusions from the obtained results while emphasizing the importance of interpreting insignificant results. Others reported difficulty in treating data sets that included missing and unclear data, extracting meaningful themes from qualitative interviews because of poor participant reports, and importing and exporting data on statistical software.

*P.5. When dealing with missing data or biased data I had to travel back and forth, which was challenging at that time due to mobility issues in the country which were exacerbated by country upheaval and war. This was one challenge. Another challenge was related to my limited experience in using SPSS. I faced technical problems regarding how to work on SPSS and how to import and export data, but I managed at the end, Thank God.*

### Manuscript write-up

While most fellows managed to finish their data collection and analysis as well as collate their findings into a manuscript for publication, some key challenges were reported. Some fellows found difficulty in situating study findings within the larger literature because of limited existing knowledge in their topic of interest. Others mentioned challenges in translating qualitative findings into tangible scientific recommendations. Another found it difficult to adjust the format of the paper to fit the journal requirements in terms of structure, content, and word count without compromising the quality of their writing.

*P.1.The journal’s format, which we chose to publish in, led me to make changes to my paper’s writing. For example, one of the requirements dictated that the reference list should not exceed 25 references, which forced me to delete multiple references because I had between 30 and 40 references in my paper initially. So yes, adapting my paper to the standard format required by the journal, in which I planned to publish, was a challenge.*

*P.4. To be honest, reporting what the participants said and interpreting it into scientific words was a challenge for me. I had to translate the participants’ spoken language into scientific words without compromising their thoughts. This was considered a little difficult for me.*

During the final phase of the manuscript write-up, numerous successes were reported by the learners. One fellow highlighted their capability to finalize research findings, successfully compile a draft, find an appropriate journal, and begin the publication process, all while amidst a global pandemic and period of political/civil unrest.

Another fellow mentioned that during the final stages of the manuscript write-up, he realized that his findings can be used on a larger scale to aid health organizations operating in conflict settings, as well as decision makers, in better understanding and considering the needs and concerns of AMR patients. He also realized that his research has the potential to form the base findings for a follow-up study that can include a larger and more diversified sample, and which can be applied within a broader context. Other fellows mentioned gaining vast knowledge of AMR which was exemplified by their ability to conduct a study within a conflict setting and write a manuscript on the topic.

*P.3. another thing of course I studied ten times the amount of information that I had on AMR during this period of time. So of course, there are the benefits of you know learning more about your topic hmmm and if at the end you know you have a paper now in your hand so this is the positive thing.*

*P.4. I believe that my research findings, even if they will not have a large significant impact, will nevertheless be useful to health organizations working in conflict zones such as north-western Syria, as they will gain a wealth of information that they can consider. This information might be able to encourage them to think about how to better serve patients, as some of my study’s findings revealed a variety of issues that patients face.*

### Long-term effects

All learners noted that long-term changes took place in relation to their research capacity, performance and behaviour, and personal development following their completion of the CREEW fellowship.

In terms of research capacity, learners noticed a significant enhancement of knowledge and skills in conducting research, specifically within the context of fragile and conflict settings and on the topic of AMR. One fellow noted that he can now conduct research in a more structured and systematic manner. Another learner reported that the research skills he developed, in terms of looking up scientific articles, allowed him to convey and share evidence-based information with government officials that is backed up by credible references. The data received from fellows was supported by information gathered from learners’ colleagues who pinpointed that the CREEW training did indeed contribute to updating the research knowledge and skills of the learners and provided them useful information on AMR in conflict settings. In addition, this fellowship allowed learners to become familiar with the possible challenges they might face when conducting research in fragile settings and conflict-affected areas.

*P.5. It was great phase okay, as an induction okay, and then impact of doing the proposal, the mentoring, having a lot of experience on using different tools of analysing the data and entering the data; different tools for citation, and how to do citation and how to use PubMed for looking into literatures. Therefore, I learned a lot of things. I never knew about how to do citations, how to do research, how to look for the proper literature, where to look for the literature. And during my looking for the literature have a great impact on my knowledge, on the researches, so yeah, I can see that the three phases have a great impact on me*.

*P.4. Of course, I attained the capability to plan for research projects specifically in the process related to receiving ethical approval from IRB or from the health authorities in the region where I worked, as well as the hospital from which the samples were taken. I also learned how to sample and how to select my samples based on the criteria and characteristics specified. Of course, I have applied the knowledge that I gained from the fellowship programme that I was attending at the ti*me.

Most notably, learners reported a change in their behaviour following the completion of the fellowship by which they were able to translate the information they learned into performance. For instance, some learners mentioned that the knowledge acquired on the methods of conducting research was applicable to their work, and for some this information was used to advance and develop the quality of their work. One learner noted that the experience gained from the fellowship allowed him to launch new scientific projects, and revise and modify those that were already implemented. One learner also mentioned that he was able to provide support and guidance for his colleagues in their research projects as a result of the experience he gained during this fellowship. Indeed, one of the learner’s colleagues did mention that the learner was “committed to the topic of AMR” and was “promoting AMR activities within the rest of the team”. At a national scale, the fellowship reportedly equipped one fellow with the knowledge and evidence that can allow him to contribute to the proposition of solutions to the healthcare system in his conflict-affected country.

*P.1 My current job, yes when it comes to study and research design because one of my responsibilities is to manage research studies and to follow on that issue. It was very helpful for me during my current job and for my academic study even because I am currently a PhD student. It was very helpful hmm in terms of research topic and knowledge and practice and in terms also of the scientific topic which is antimicrobial resistance, so it was really a great experience and helpful in my current duties and responsibilities.*

*P.5: I am practicing it every day, as I told you we used all the researches on the antibiotic all the data, all the… to launch another antibiotic stewardship in another project, and also restart all the data, committee and all the programme of the rational prescriptions in Abs hospital one of the biggest hospitals covered by MSF in Yemen, so I was having direct relation to the job to work and also improve the quality of care of the patients directly. So, as I told you we have antibiotic stewardship in Abs and now we started another antibiotic stewardship in Kanawa and now I am applying the same tools of collecting the data, data analysis based on the results of the research that I did with the CREEW.*

All learners reported that development took place on a personal level as well. For example, some learners mentioned that the fellowship positively affected their career path and their credentials as researchers. Others noted that they gained the knowledge, skills, and confidence to work towards becoming established researchers capable of conducting further research in the future. Two learners mentioned that they were able to benefit from the fellowship in their postgraduate studies where they applied the principles learned to carry out their own research. Some learners also managed to disseminate their research findings at health conferences and in the form of publications; and while not all learners were able to share their work in the given timeframe, they all expressed their enthusiasm to do so and to become involved in future research activities.

P.4. *Today, as I began working on the write-up of my thesis, CREEW training served as a reference for my work which also allowed me to support my peers when it comes to working on their research studies. I was able to do this because I learned the research principles from CREEW; those principles also served a basis for my research studies at the university level specifically for my Master’s thesis in political science and international relations.*

P.1. *For the journal publication, I am working on that for the publication of the paper, for the dissemination workshops yes, we have presented in at least two or three webinars, one was locally and the second was through GHI webinar and the third one was, the third one was related to a network or a forum supported by the GHI and university in East Anglia, the UK, so yes, we did the dissemination and working on the publication*.

### Mentorship and networking

The significance of this programme was clearly expressed by fellows. Regarding their learning experience, fellows considered it a worthwhile and beneficial experience, noting that contextualization of the fellowship to conflict settings added value to the programme. The fact that this programme was completed by AUB, a reputable university in the MENA region, added to the significance of the learning experience as well. In addition, learners expressed their appreciation for the continuous support provided by the CREEW team and the follow-up process, which allowed fellows to provide feedback regarding the programme.

P.1. *The programme’s overall strength and distinctiveness, as well as the courses’ and mentorship guidance’s uniqueness, are built on their focus and specialized customization to the conflict setting. I’ve worked with a variety of fellowship and mentorship programmes, but what sets this one apart from the rest is that it’s specifically designed for conflict settings. This is the programme’s most major strength.*

P.5. *Even when I am telling you the nature of my work and even the context of Yemen as you know we are working in a conflict area, and working with MSF, you know what MSF is, we work in the frontline, so it was not easy for me. However, the CREEW team was continuously following up and this is something that I really admire, even at the time that I feel low because maybe I cannot continue so no they were continuously supporting me; they were even offering help for me. So the thing is that the continuous support that I got and that I am still getting is amazing, so they keep pushing you, not push you, support you and to do the things. It was a great experience to be honest.*

As reported by the fellows, the networks and connections fellows formed were important aspects that contributed to the significance of the programme. Participants noted that being in contact with fellows from diverse backgrounds and countries enriched their learning experience and enhanced the exchange of information. It also allowed them to expand their network as researchers in conflict settings. Learners also mentioned that they became better acquainted with health professionals through their research projects and were put in contact with qualified and competent instructors.

P.1. *In addition to the presentation of multiple disciplines, another significant feature of this programme is that it combines multiple fellows who are either interested in conducting research across the different MENA countries with a similar conflict setting context, or who themselves reside in conflict settings. For instance, I am from Palestine, someone was from Yemen, another from Syria and there was also someone from Iraq. This combination was regarded as a unique and strong point given that it facilitated learning by the ability to share ideas and being exposed to ideas coming from others.*

P.3. *I think the one thing that I did do is I immediately took the programme forward with people who were interested in medicine, health and public health. I introduced them to the programme. I really wanted to replicate it, to find a way to replicate it here with people who are on the field. You see, from my experience I came into contact with people who were really interested in what’s going on, and I found out if you want to do research in areas that are still witnessing conflict, these are the really really tough zones, you need to have people partners in those areas. Hmm, whenever I would talk to them about what I was doing with my research, they got very interested, and they really wanted to find a way to even themselves get involved in the programme.*

The mentorship component of the fellowship was considered a unique and excellent experience by all learners. Participants reported that beyond the theoretical aspect of the fellowship, the mentorship phase provided them with practical experience of conducting research; fellows also noted that the rigorous guidance and support provided by the mentors allowed them to overcome challenges that arose during project implementation. Learners showed great appreciation for their mentors, whom they described as experienced, knowledgeable, capable of conveying information clearly, and who reportedly demonstrated flexibility when needing to conduct meetings with learners. The mentorship experience was also considered valuable by learners’ colleagues, with one of them mentioning that the direct learning received from mentors contributed to the acquisition of knowledge and skills in the field of research. This colleague also added that this experience wouldn’t have been “optimal” had it not been followed by this phase.

P.1. *The mentorship as I understood it mainly helped to guide the research project, so regular meeting with the mentor was very helpful to speak about the challenges to overcome the obstacles and to discuss any possible alternatives for any obstacle that hindered the process. So hmm I think it was very helpful having a mentor that can guide the process rather than doing or replicating the role of teaching or providing scientific information. It was about how to move with the research.*

P.3. *And after the mentorship, I think I am a lot more. Well, I learned how mentors, how a professional mentor really handles topics. And also I got to know a good writer, you know my mentor is a researcher and I got to read a lot of research that he does, and so I think that is important also, I think if you have a good mentor and you can read what they’ve written, and you have access to their papers, they’re a mentor with you for, they’ll stay with you, you know the experience is more prolonged because you can always go back and study what they told and you can study what was written.*

Despite the positive overall experience reported by learners, some fellows faced certain challenges that were related to logistical and external factors. One learner in particular reported issues with accommodation during the didactic phase which was completed in Beirut, noting that they didn’t receive their stipend on time, which further impeded their ability to continue their research project. Another learner mentioned that the limited funding received was not enough to support their research project. Additionally, some learners reported that the time allocated for project implementation was insufficient, especially among those with other work obligations.

Furthermore, it was challenging for fellows to cope with the emergencies that occurred within their conflict settings while completing their fellowships, particularly with the advent of the COVID-19 pandemic which further imposed limitations on learners’ mobility. Indeed, one of the learner’s colleagues did mention that the COVID-19 situation imposed barriers on the learners and limited their ability to collect data and their access to research laboratories. Lastly, one learner was not able to complete the research project after needing to change the topic due to contextual restrictions set by the pandemic; this fellow believed that their educational background was incompatible with the newly assigned project and therefore opted to terminate the research project.

P.1. *A general shortcoming is the restricted resources available during the fellowship process; the financial resources that were available do not allow you to perform a large-scale project. If you want to conduct a high-quality experimental project, you’ll need a large budget, but the budget we had was considered small.*

P.4. *As a humanitarian worker, I found out that I didn’t have enough time to work on my research project. Now that CREEW has been in operation for almost two years, I believe the time allocated to complete the research study was insufficient. For instance, I could not find the free time to conduct interviews, analyse the data and perform transcription.*

## Discussion

The present study evaluated the CREEW-AMR fellowship, which aimed to equip frontline health workers in fragile and conflict-affected settings in the MENA region with the skills to conduct research on AMR in the context of war. Given the scarcity of previous research on similar topics globally, and limitations in this area of research identified in a previously published scoping review conducted by GHI on HRCB highlighting the need for more carefully designed evaluations, this study adopted a qualitative longitudinal design. This study design was essential given that the prior scoping review specifically emphasised the need for long-term evaluations that involved data collected not only at the individual level but also at the level of the organisation, institution, and/or system [18]. Fellows were followed across multiple time points throughout the 3 phases of the fellowship programme; namely phase 1 of online and in-person courses/seminars, phase 2 of field research (conception to manuscript write-up), and phase 3 of research dissemination. A total of 5 fellows participated in the programme, 4 of which succeeded in producing research outputs, and 1 of which did not manage to produce originally intended research output.

To the best of our knowledge the present study is the first such evaluation of such HRCB programmes in conflict-affected settings in the MENA region and adds significant value to the literature by virtue of its design. The main strengths of this study include the in-depth exploration of fellows’ experiences throughout the phases of conceptualizing, designing, and implementing their research projects. In turn, this allows for enhanced understanding of key successes and challenges encountered by fellows in such a training programme. This study also has strong potential to inform the future implementation of similar programmes, which are likely to increase over time.

Findings generally indicate that the overall design and modalities of the CREEW fellowship were well suited to achieve the set objectives. That is, courses offered prior to research field work were found to be crucial to equip fellows with knowledge on research methods in conflict settings, along with a solid understanding of the chosen thematic topic. Although preference for in-person courses / seminars were clearly articulated by fellows, an appreciation of transitioning to online modality was also reported as a valuable alternative to overcome transportation, security, financial, and logistical barriers. Indeed, e-learning has been noted as an efficient and effective tool to enhance accessibility and availability of training opportunities in low-resourced settings [35], yet it also runs the risk of socially isolating learners [36]. This was re-iterated among fellows where in-person courses were perceived to be more engaging and interactive compared to online courses which were found to be more practical and accessible given the circumstances. In view of the present focus on conflict settings, both modalities warrant consideration in future programmes. There is value in prioritizing in-person contact - even if partially - throughout the fellowship, and in complementing it by setting adequate infrastructure for online offerings with special attention placed on engagement strategies and interactive features [37]. This is because in-person attendance may pose financial, security, and logistical challenges to attending fellows, which may compromise their completion of the fellowship should an online component not be considered. At the same time, online components can benefit from more thoughtful and careful planning to keep fellows engaged with instructors and colleagues. Importantly, because the programme enrolled a limited number of fellows, it was possible to maintain strong engagement with them especially given the close ratio of mentors to fellows who were expected to work very closely together. This is also a crucial point to consider in similar programmes.

As for the research phases, including proposal development, IRB application, data collection, data analysis, and write-up, a range of challenges and successes were identified. For instance, fellows reported difficulty in applying knowledge gained from the courses when developing their proposals, including research design considerations and technical knowledge on AMR. This was further aggravated by them having to place additional efforts on adapting their projects to better fit constraints in their home countries imposed by conflict-related challenges, and to prepare contingency measures should unexpected events occur. This is an important finding because it characterizes the typical experience of many researchers in conflict settings such as struggling to identify adequate and updated literature on topics of interest, difficulty developing a data collection plan, factoring-in political and security risks, considering social resistance, and difficulty in obtaining multiple permits from multiple sources, among others [2, 20]. Therefore, such insights are critical lessons that need to be communicated to fellows, alongside practical steps on how to overcome them. Another noteworthy challenge reported prior to data collection was the process of applying for ethics approval. Fellows generally expressed concerns regarding finding institutions to apply to, waiting long periods for revisions, and the potential delays their projects may face as a result. An additional layer of complexity was added for projects that targeted human subjects in view of the sensitive nature and high ethical risks involved in that type of research. Understandably so, conflict settings are ridden with ethical, social, and political issues that make up part and parcel of important considerations when undertaking research activities.

As for actual field work, several challenges were identified on multiple levels. This includes the general lack of research culture and support for research initiatives, which made it difficult for fellows to form collaborations, obtain secondary data from authority figures, and receive required permits. Also, some fellows experienced difficulty managing data retrieved from some institutions and communicating with others due to limited expertise among local partners. Furthermore, movement restrictions due to security risks (e.g., armed conflicts and natural disasters such as flooding) and the COVID-19 pandemic, limited ability to access conflict zones, and limited ability to capitalize on technology, all impacted data collection in terms of timeline, quality, and quantity. Finally, psychosocial and cultural considerations were also important challenges when collecting data such as ensuring gender balance and representation. Post field work, minor challenges were reported such as having to clean and treat data sets, spending much time on transcribing and translating qualitative interviews, aligning data analysis with the aims of the study, and formatting manuscripts to fit journal requirements. Many of these aspects might not have been learned by theoretical or didactic pedagogical approaches alone, whereby practice and mentorship are necessary to optimize learning outcomes, further justifying the importance of this fellowship in such contexts and for such populations.

Despite all aforementioned challenges, which are expected to be encountered when conducting research in conflict settings, all but one fellow managed to successfully produce originally intended research outputs and submit them for publication or conference presentation. In general, all fellows reported increased knowledge on research methods and AMR, increased confidence and capacity to design, implement, and disseminate research, and better understanding of the role of research in their service as frontline health workers. For instance, fellows reported learning how to formulate research questions, design appropriate methodologies in alignment with their research goals, develop tools, apply for IRB approval, coordinate with local partners, collect data, analyse data, and translate findings into practical recommendations that have practice and policy implications. Despite expected difficulties encountered, these objectives were achieved, and therefore the programme successfully managed to achieve its goals. These findings were also corroborated by data collected from peers and supervisors of fellows.

Throughout the fellowship, fellows were paired with a mentor who supervised their work and advised them across all phases of the research. This is a crucial aspect of the fellowship because while fellows conducted the work independently, they had close contact with experts in the field. So, the multiple challenges reported above were largely overcome by collaborating and communicating with respective mentors, and this ultimately provides an ideal approach to encourage learning and skill development given the necessity of fellows to overcome adversities and obstacles while having adequate guidance. The modality adopted in this fellowship also contrasted barriers traditionally observed in remote learning such as maintaining engagement and reducing isolation, since the core focus was on pairing mentors and fellows to work closely together on achieving set research goals. The importance of having mentors to guide learners in such programmes has been previously highlighted in several studies, as it was reported that connecting peers with mentors motivated participants to complete their assignments and fostered the development of collegiate connections [38, 39]; whereas the absence of a mentorship delayed the implementation of the recommended research activities [40]. Indeed, this was potentially the main contributor to the programme’s success, along with the presence of a committee of experts who could be consulted on various matters.

## Limitations

Despite the value that this evaluation adds to the literature, it is important to interpret results in light of several limitations.

First, this programme was largely affected by COVID-19 related restrictions which can be considered exceptional circumstances that impacted the work of fellows and their attendance to in-person seminars, and subsequently the quality of data collected. That is, longer than expected delays were noted throughout the field research phases because of the pandemic and other challenges which caused multiple deviations from data collection timeline, especially considering that fellows had different research designs. While some had to collect primary data from human subjects or lab samples, another received secondary data, and another conducted a literature review; this meant that periodically collected evaluation data could not be standardized across timepoints. Still, this experience did not impede the completion of the fellowship, as all fellows managed to produce said research outputs.

Second, our sample was relatively small and quite heterogeneous, considering differences in fellow’s backgrounds, skill levels, country of residence, and research design. This meant that much variability was observed across fellows whereby data saturation may not have been reached. Nevertheless, their narratives still provide important considerations for designing, implementing, and evaluating such programmes, and provide some foundation for future programmes to build upon. That said, it is important to recognize that such programmes may prove difficult to coordinate with too many fellows enrolled, and may prove to be less engaging for fellows should the ratio of mentors to fellows be too large. Also, having a smaller sample size means that the programme can be more tailored and fellow-centred and can result in a deeper and richer learning experience for each individual fellow. Larger programmes enrolling a larger number of fellows may require significantly more funding to account for increasing costs for hosting institutions, staff and coordinators, mentors, research equipment and related expenses among others to guarantee rich learning experiences and smooth coordination.

Third, our evaluation might have missed essential information regarding acquisition of skills and behaviours during and after the fellowship as we relied primarily on subjective reports with little objective measures beyond achievement of research outputs. Our evaluation could have also benefited from further triangulating data with mentor perceptions. Future research could focus on this aspect of applying more objective assessments for knowledge and behaviours and on incorporating the experiences of mentors given their central role in such programmes.

Finally, our evaluation was conducted at the level outputs and outcomes. Future programmes should aim to evaluate such programmes on the longer term at the system level of policy and health impact since that would be the ultimate goal of implementing such fellowships.

## Conclusion & recommendations

This evaluation primarily aimed to evaluate the model applied in the CREEW fellowship. Although the focus of this cohort was on AMR, this model can be adapted to focus on any health-related topic and has strong potential for replicability and scalability. In general, we found that this model is well suited for application in conflict settings, given its reliance on in-person and online modalities, heavy focus on remote mentorship throughout all research phases, pairing fellows in conflict settings with mentors from the high and low-income countries, and provisional granting of the certificate pending production of research outputs. These conditions create an ideal climate for fellows to (1) learn the foundations of research in conflict settings and essential background knowledge on their topics of interest, (2) have regular guidance and support from the hosting institution and mentors when conducting research, and (3) work towards producing a tangible research output.

Furthermore, our reliance on a longitudinal qualitative design allowed our analysis to yield in-depth understanding of the fellow’s experiences on a small and select sample operating from 5 conflict-affected countries. Importantly, we were able to identify specific successes and challenges, and to capture potential opportunities that other programmes may benefit from in similar programmes across each of the key research phases. The field of HRCB has only recently surfaced as an important approach to improve health research production in conflict settings and more efforts should be directed towards investing in such programmes because of their value in preparing frontline health workers to produce research on the relationship between health and war, and to conduct appropriate evaluations. Ultimately, this is key to improving health policies and health outcomes in conflict settings in a context where research is primarily dictated by influences from higher income countries, lacking contextualization to local considerations, and lacking local leadership.

Based on our findings, we recommend the following points to be considered in future similar programmes:

- Design.
  - Ensure that the design of the fellowship includes in-person and online modalities with a content that capitalizes on practical and engaging approaches. Remote mentorship is also critical to the success of such programmes.
  - Consider incorporating real-life examples and information that covers actual complexities of conducting research in conflict settings such as navigating political and administrative environments, special considerations when working with target populations among others. Many of the available material on research methods and on chosen thematic topics may need to be contextualized to the region of operations and this is why having local leadership in course development and delivery is key.
  - Allow room for flexibility in delivery of course material based on the needs of fellows. It is likely that enrolled fellows will represent diverse educational, cultural, professional backgrounds, which the programme may need to account for.
  - As a prerequisite to enrolment, make sure that selected fellows have adequate knowledge in thematic topic, some experience and knowledge of research in conflict settings, a solid and grounded proposal, a strong justification for applying to the fellowship, good English writing and verbal skills, and some flexibility in their schedules. Based on our experience, these criteria may be important predictors of performance and commitment to the fellowship.
- Implementation.
  - To maintain engagement with fellows, which is a common obstacle in online and remote learning modalities, it is crucial to ensure a close ratio of mentors to fellows as a means to ensure greater tailored, continuous, and focused support.
  - Hosting institutions are encouraged to consider setting up collaborations and agreements with labs, hospitals, IRBs, and other forms of authorities as part of their programmes in order to facilitate coordination and communication and thereby reduce delays and preventable complications.
  - Given the context of conflicts, contingency plans of reserve funds, deadline extensions, alternative communication methods, alternative course delivery methods, and flexibility in adapting research designs should also be considered and potentially implemented throughout the course of the programme should the need arise.
- Evaluation.
  - Set up systems for monitoring and routine collection of data from each fellow across the research phases. Each research phase represents a milestone in the fellowship programme, to which fellows may have different experiences, outcomes, and challenges, and these are important to capture in the evaluation.
  - Qualitative assessment might be more appropriate forms to evaluate such programmes given the small sample sizes of enrolled fellows and the higher need for in-depth exploration of experiences.
  - For larger programmes, consider setting up mechanisms for system-level evaluations such as impact assessment of research outputs on skills acquisition, health policies, and health outcomes.

## Electronic supplementary material

Below is the link to the electronic supplementary material.

Supplementary Material 1

Supplementary Material 2

Supplementary Material 3

## Data Availability

The datasets generated and/or analysed during the current study are not publicly available due lack of ethics and participants approval to publicly share datasets that are in-depth qualitative interviews. However, the datasets are available from the corresponding author on reasonable request. .
