# Supplementary material for: Evaluating a research training programme for frontline health workers in conflict-affected and fragile settings in the middle east
Source: BMC Med Educ. 2023 Apr 13;23:240. doi: 10.1186/s12909-023-04176-6 (PMC10099017; doi:10.1186/s12909-023-04176-6)
Supplement: Supplementary file 1 — Supplementary Material 1 [file 12909_2023_4176_MOESM1_ESM.pdf]

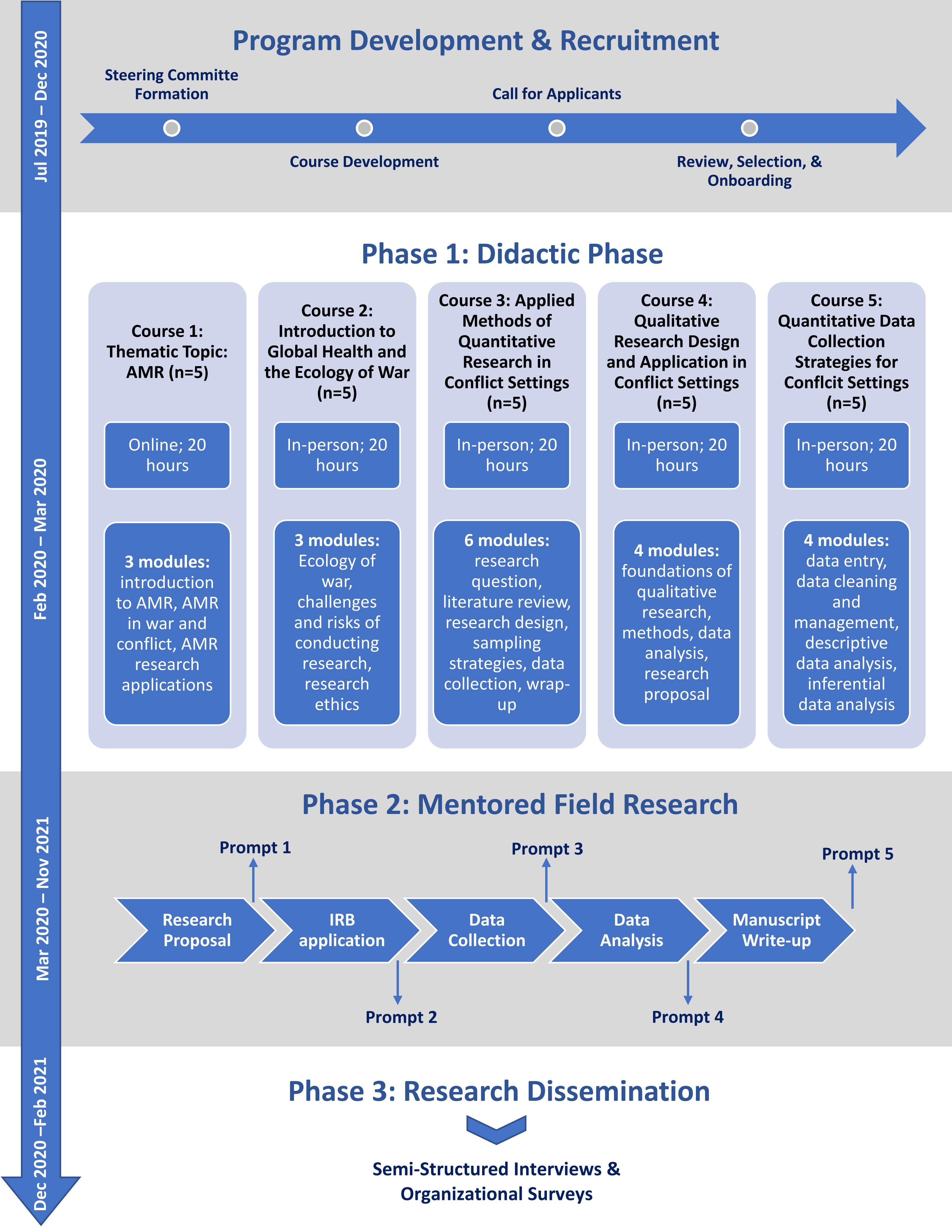
Jul 2019 – Dec 2020
Program Development & Recruitment
Steering Committe Formation
Course Development
Call for Applicants
Review, Selection, & Onboarding
Feb 2020 – Mar 2020
Phase 1: Didactic Phase
Course 1: Thematic Topic: AMR (n=5)
Online; 20 hours
3 modules: introduction to AMR, AMR in war and conflict, AMR research applications
Course 2: Introduction to Global Health and the Ecology of War (n=5)
In-person; 20 hours
3 modules: Ecology of war, challenges and risks of conducting research, research ethics
Course 3: Applied Methods of Quantitative Research in Conflict Settings (n=5)
In-person; 20 hours
6 modules: research question, literature review, research design, sampling strategies, data collection, wrap-up
Course 4: Qualitative Research Design and Application in Conflict Settings (n=5)
In-person; 20 hours
4 modules: foundations of qualitative research, methods, data analysis, research proposal
Course 5: Quantitative Data Collection Strategies for Conflcit Settings (n=5)
In-person; 20 hours
4 modules: data entry, data cleaning and management, descriptive data analysis, inferential data analysis
Mar 2020 – Nov 2021
Phase 2: Mentored Field Research
Research Proposal
IRB application
Data Collection
Data Analysis
Manuscript Write-up
Prompt 1
Prompt 2
Prompt 3
Prompt 4
Prompt 5
Dec 2020 –Feb 2021
Phase 3: Research Dissemination
Semi-Structured Interviews & Organizational Surveys
