# Supplementary material for: Evaluating a research training programme for frontline health workers in conflict-affected and fragile settings in the middle east
Source: BMC Med Educ. 2023 Apr 13;23:240. doi: 10.1186/s12909-023-04176-6 (PMC10099017; doi:10.1186/s12909-023-04176-6)
Supplement: Supplementary file 2 — Supplementary Material 2 [file 12909_2023_4176_MOESM2_ESM.docx]

Appendix – Themes from Qualitative Interviews

SSI and Prompts Combined

|  | **Theme** | **Codes** |
| --- | --- | --- |
| **Course Delivery** | **Learning Modality** | - Online learning was a new experience - Although learners are becoming more accustomed to online learning due to the COVID pandemic, in-person learning preferred over online learning - In-person learning allows for better understanding and grasping of information - In-person learning allows for better engagement and interaction through allowing role play in exercise - Online learning is self-directed and requires more organization and discipline, rendering it more difficult - Online learning allows learner to refer back to material as many times as needed to understand the material - Online learning can be useful in times of COVID - Online modality did not pose any technical problems |
|  | **Syllabus** | - Combined theoretical and practical aspects of research - Courses were multidisciplinary and provided learner with the basics of conducting research in conflict settings and with fundamental information on AMR - Modules were clear, well-prepared, and enhanced learning experience - Courses provided credible information and resources - Instructors were regarded as knowledgeable and experienced within their field - Assignments enhanced engagement with fellows and were contextualized to fragile and conflict settings - Evaluation questions allowed learner to self-assess learning |
|  | **Challenges** | - Courses were not completed properly due to the COVID situation which forced learners to move back to their countries - More time was allocated for basic information; whereas more important information was not covered properly - Additional time needed to cover some course material properly; specifically courses related to statistical analysis |
| **Proposal Development** | **Successes** | - Development of the proposal on time, presenting it in front of a panel and being able to get feedback in return was positively perceived - Acquisition of knowledge and increased understanding on how to structure proposals and on the procedures involved in producing research - Obtained knowledge and experience on how to put a research objective, how to design a research question, and how to choose a design - Forming a good relationship and receiving support from hospital management and personnel - Gaining a new perspective and a deeper knowledge on AMR - Proposal and methodological modifications suggested by committee members were regarded as a beneficial experience to learn from |
|  | **Challenges** | - Limited literature on the topic of AMR in Africa or in conflict zone - Challenges faced in accessing online library - Uncertainty on how to integrate the quantitative and qualitative methodology in the research design of the study - Discrepancy between the initial theoretical understanding of research and the research principles encountered in practice - The need to transition from one research plan, design and methodology to the other while developing the proposal - The shift of the objective of the proposal to medical was regarded as incompatible with the learner’s profession and with the primary initial objective - Learner’s belief of lacking the necessary qualifications to continue with the project - Learner’s lack of experience in communicating the needed equipment with the lab - Lack of coordination between labs in site of research from one end, and learner’s mentor on the other end - **Subtheme: Contextual Challenges** - The limited mobility imposed due to COVID-19 posed challenges in accessing facilities to plan for data collection - Covid-19 and conflict setting impacted the ability to access the ministry of health and get information from it |
| **Ethical Approval** | **Successes** | - Gaining knowledge on how to get IRB clearance from ministry of health - Forming a rapport, and obtaining positive feedback and support from the personnel of the IRB office - Increased knowledge regarding the different IRB application forms and the required documents - Knowledge gained on the process of obtaining ethical approval that is contextualized to conflict settings |
|  | **Challenges** | - Limited knowledge of where to apply for the IRB application - Difficulty in adjusting the IRB based on the comments received - Concern of not receiving IRB approval following the modifications and efforts - Getting permission from the hospitals to obtain samples was hindered by the leaner’s limited experience in research and medicine and by the hospital’s lack of readiness for this procedure - Period of unrest in country impeded the ability to find a university from which the IRB approval can be obtained - Obtaining IRB approval was difficult and time-consuming, especially during COVID |
| **Data Collection** | **Successes** | - Understanding the role of ethics in constructing the research and the role of questions asked during data collection on impacting the results - Ability to obtain required data from the target sample during times of pressure and country unrest - Ability to diversify the sample and recruit female participants - Gained knowledge on how to conduct and manage interviews - Increased ability to use proper probing when conducting interviews |
|  | **Challenges** | - Non-representative samples that necessitated resampling - Credibility of the target sample in terms of the required characteristics (AMR + or -) - Limitations related to technicalities in the sampling procedure in terms of quality assurance and the absence of substances that were needed for sample testing - Disapproval of some of the participants to participate in the study after receiving the consent form, although they initially agreed to participate before receiving the form - Difficulty in recruiting and reaching out to participants because some of the AMR patients’ contact numbers were missing in the hospital or required travel across borders - Recruited participants exhibited limited understanding of the content and the objective of the interview questions, thus extending the duration of the interviews - Experiencing emotional turmoil while conducting interviews with participants living in war and conflict areas - Successful testing of samples in the hospitals was inhibited by the obtainment of unreliable test results due to the unqualified and uncooperative hospital labs, and improper communication between the lab and the doctor (mentor)s - Technical issues regarding internet connectivity impeded the conduction of the online interviews and the scheduling procedure - Transcription of the interviews took much time and effort to complete especially for long interviews - **Subtheme: Contextual Challenges** - Learner’s country being in conflict and the impact of unrest on the health system and ability to collect and store data - Restricted mobility across country borders during times of Covid-19 to recruit the samples from the hospital - Difficulties in transportation and visiting the hospitals for the sampling procedure due to Covid-19 restrictions |
| **Data Analysis** | **Successes** | - Ability to complete data analysis and present it amidst the COVID pandemic - Ability to transcribe all the interviews despite time restraints - Incorporating successful procedures to organize and prepare the data for completing the data analysis and producing significant results for the study - Obtained skills to conduct data analysis and be able to extract codes and themes - Obtained knowledge on how to use statistical analysis software (SPSS) and understand the visual outputs of the results |
|  | **Challenges** | - Difficulty in extracting the themes from the interviews considering the lack of depth in information provided by the participants - Technical difficulties with importing and exporting data on SPSS - **Subtheme: Contextual Challenges** - The COVID-19 measures forced the universities to close and stopped in-person meetings which hindered the process of data analysis - Difficulty in restoring missing data from hospitals and managing biased data due to restricted communication, transportation, and mobility in a war setting |
| **Manuscript Write-Up** | **Successes** | - Despite the challenging period and the global pandemic, the learner was able to progress with the study, finalize the research findings, and the write-up, and start with the publication process - Ability to choose a proper and a relevant journal to publish the paper |
|  | **Challenges** | - Limited literature to support the research findings and the discussion section due to the uniqueness of the topic and the need to target the context and conflict setting - Difficulty in transforming the responses of the participants into scientific terms - Having difficulty summarizing and condensing the article to fit the word count requirements without compromising the content of the research - The need to modify the paper as per journal’s submission guidelines - Termination/discontinuation of research study due to the choice of topic being outside the scope of the learner’s profession and capabilities - **Subtheme: Contextual Challenges** - Need to restructure the planning, reschedule deadlines, and re-manage the write-up process due to contextual difficulties and the COVID pandemic |
| **Long-Term Changes** | **Research Capacity** | - Acquisition and enhancement of knowledge and skills related to the research process and AMR - Developing skills that allowed sharing of information that is backed up by scientific evidence and credible references - Learner became familiar with the possible challenges researchers might face when conducting research - Unique focus on fragile and conflict settings enhanced learner’s skills in conducting research that is specific to conflict regions |
|  | **Translation of Learning into Performance** | - Knowledge on research process acquired from fellowship was applicable to, and implemented in learner’s work - New perspectives gained on data interpretation allowed learner to advance the quality of his work - Experience gained from fellowship allowed learner to launch new projects and revise projects that were already implemented - Knowledge and skills gained during fellowship allowed learner to provide support and guidance for his colleagues in their Master’s research - Enthusiasm shown to be involved in future research activities and events through projects and conferences - Dissemination of research findings in health conferences and through publications - Contributing to the proposition of solutions to the health system of a conflict-affected region - Eagerness to replicate CREEW fellowship program in learner’s country |
|  | **Personal Development** | - Knowledge and skills gained play a role in impacting learner’s career path and personal development - Gaining the knowledge, skills, and confidence to conduct future research and consider oneself as a researcher - Fellowship experience complemented learner’s post-graduate education - New experience in conducting research and noticeable increase in research knowledge |
| **Significance of the Program** | **Learning Experience** | - Fellowship was contextualized to research in conflict settings specifically, which added to the uniqueness of the program - Despite the high intensity of the program, learner considers it a worthwhile and beneficial experience - Learning experience from a reputable university, such as AUB, was well received - Learners expressed appreciation for the continuous support provided by the CREEW team - Appreciative of the follow-up conducted and his ability to give feedback on the program |
|  | **Networking and Connections** | - Being in contact with fellows from diverse backgrounds and countries enriched the learning experience and the exchange of information - Fellowship allowed learners to expand their network and connections in the research field of conflict settings - Mentorship allowed learner to become acquainted with health professionals - In-person courses provided learners with contact to qualified and competent instructors - Learner is still in contact with his mentor and maintains a positive relationship with him |
|  | **Mentorship** | - Described as a unique and excellent experience - Provided practical experience on conducting research - Mentors’ suggestions provided proper guidance for learners and allowed them to overcome challenges - Mentorship gave learner access to articles written by the mentor himself, which allowed the learner to learn from them - Mentors were knowledgeable and experienced with conducting research in conflict settings - Mentors provided constant support and encouragement and followed up with learners at every phase of the research and during times of conflict - Mentors were responsive and demonstrated flexibility and readiness to conduct meetings at any time - Information and instructions communicated by mentors were conveyed in a clear and concise manner - Interaction with mentor was well maintained through online platform |
|  | **Challenges** | - Organizational problems were faced; such as receiving stipends on time and issues with accommodation - Not receiving a stipend impeded the ability to continue with the research project and recruit a team - Limited funding resources to support the learner’s research topic - Coping with emergencies that arise from conflict settings added to the challenge of completing research-related tasks - Time allocated for project implementation was not sufficient, especially for those who are working in parallel or are in the process of completing postgraduate studies - Learner considers that selection of fellows should have been based on more precise and clear criteria, considering that his field of work does not match the fellowship’s topic - Learner’s knowledge not compatible with research requirements considering that he does not have a scientific background - Learner unable to complete his research since it did not match his field of work |
