# Supplementary material for: Evaluating a research training programme for frontline health workers in conflict-affected and fragile settings in the middle east
Source: BMC Med Educ. 2023 Apr 13;23:240. doi: 10.1186/s12909-023-04176-6 (PMC10099017; doi:10.1186/s12909-023-04176-6)
Supplement: Supplementary file 3 — Supplementary Material 3 [file 12909_2023_4176_MOESM3_ESM.docx]

Appendix:

**Programme Development**

CREEW was developed with the aim of equipping frontline health practitioners, working in conflict-affected settings with the necessary skills that would enable them to conduct research into the relationship between health and war. The primary focus of the fellowship was to encourage a culture of research in settings where research is underprioritized. A steering committee composed of six members, who are renowned clinicians and researchers in conflict settings, was formed in order to set the strategic vision and guide the long-term trajectory of CREEW. This committee was co-chaired by the director of GHI and the Swisscross Foundation - a partner organization that financially supported CREEW- and included two members from GHI and two other members from the Swisscross foundation. In addition, a Scientific Advisory Committee (SAC) composed of six members was established and included two co-chairs and four members, all of whom are experts in conflict medicine. The SAC was responsible for selecting the thematic topic, advising on the selection of subject matter experts and mentors, and consulting regarding the course materials and research process. The SAC was also responsible for overseeing the mentored field research and dissemination process as well as interviewing and selecting candidates. Furthermore, an admissions committee was formed before the application start date and it was composed of six members including the co-chairs of the SAC, the Steering Committee, the GHI Academy Manager, and the CREEW Coordinator (nonvoting).

Depending on the thematic topic, a list of potential mentors was suggested by the SAC prior to the application deadline. Mentors were required to have prior experience working in a given health field, and in a conflict setting. They should be established researchers with a solid and proven understanding of conducting research in conflict settings (i.e., including knowledge of security and ethical risks involved with health in conflict research). Mentors were assigned based on their research areas of expertise and were matched with fellows accordingly. They were responsible for guiding fellows in developing their research projects, implementing their data collection field work, and producing the final research output. Mentors met frequently with their assigned fellows throughout the course of the programme virtually. Additionally, they had to ensure that the fellow delivered the field project proposal in a timely manner and were required to update the SAC on the fellow’s progress.

**Thematic Focus: Antimicrobial Resistance**

The first cohort’s area of focus was Antimicrobial Resistance (AMR) in conflict settings. AMR has been highlighted as a leading cause of death in the 21^st^ century, with recent estimates indicating it could kill 10 million people per year by 2050.^11^ An extensive review by the Antimicrobial Resistance Collaborators was recently published in The Lancet quantifying the impact of AMR on the global population; they concluded that approximately 4.9 million deaths were associated with drug resistant infections, and 1.27 million deaths were directly attributable to drug resistance in 2019 alone.^12^ Their data indicated that AMR presented a particularly higher burden in LMICs, with all-age death rates highest in some LMICs. The researchers suggest that this high burden is due to the scarcity of laboratory infrastructure, inappropriate use and insufficient regulation of antibiotics, ease of acquisition of antibiotics, substandard antibiotics that drive resistance, and poor sanitation and hygiene. The authors also note that high-quality data on infectious disease, pathogens, and AMR was only sparingly available in LMICs. Another recent review focused specifically on bacterial infection during wars, conflicts, and disasters in the MENA region found that drug-resistant infection outbreaks have not only surfaced as a direct outcome of war but are also worsened by poor hygiene and sanitation, destroyed health infrastructure, and the displacement of refugees.^13^ This review also highlighted the impact of traumatic war wounds on the development of infections where important driving factors for pathogen survival include: time to access care, onset of antimicrobial agents, and environmental contamination.^13–15^ As such, AMR presents a crucial health threat among FCAS in the MENA region, where conflict and fragility have severely impacted the health services needed to understand, prevent, and overcome the burden of AMR.^16^ It was thus fitting to have the first cohort of the CREEW Fellowship study, explore, and research AMR as it applies to their specific contexts.

**Programme Design & Delivery**

The Fellowship programme adopts uniquely engaging learning modalities including an online course, in-person training, and virtual mentorship, all of which are applied under the three phases of the fellowship: (1) a didactic phase (see table 1 for overview of courses), followed by a (2) field-based mentored research phase, and culminating with a (3) research dissemination phase. Each fellowship cycle spans 24 to 36 months and is focused on a context-specific health challenge that is determined annually. The first cohort of learners successfully completed the CREEW Fellowship in March 2022.

*Phase 1: Didactic Phase (February 2020 - March 2020)*

This phase consisted of four courses focused on strengthening research skills, and a thematic course on AMR. The first course was delivered online and the remaining four courses were carried out in-person at the American University of Beirut. Courses were both didactic and practical, with emphasis on conducting research in conflict settings. Courses were developed in June 2019 and delivered by Subject Matters Experts (SME) in their respective fields for researchers below Master’s level credentials. The courses primarily focused on teaching fellows how to set up data collection systems and supervise data collection processes as part of leading on research studies. This would in turn prepare the fellows to manage the data and analyse them alongside their mentors. A variety of pedagogical approaches were implemented during the sessions, including: interactive lectures, individual activities, debates and discussions, case studies, demonstrations, and role plays. The duration of each approach ranged between 10 and 120 minutes, and a total of 120 hours were delivered over 65 sessions. The passing grade was 60%, and grades were awarded based on attendance and performance for each course.

CREEW’s on campus courses began on February 26, 2020 and were expected to last until March 18, 2020. However, due to the COVID-19 outbreak, the courses had to be cut short and were therefore terminated a week earlier, on March 11, 2020.

*Phase 2: Mentored Field Research Phase (March 2020 - November 2020)*

Following the didactic phase, CREEW fellows conducted field research on the thematic topic over a period of 9 months. CREEW’s mentored field research began on March 26, 2020 with the research proposal vetting workshop, which was held online due to the outbreak of the COVID-19 pandemic. During the workshop, fellows presented their proposals to the SAC and received feedback and recommendations to modify their protocol, before finalizing it and submitting their IRB application. After passing this stage, fellows proceeded with collecting data and preparing their datasets for analysis in the third phase. Throughout the research phase, fellows maintained regular communication with their mentors.

*Phase 3: Research Dissemination Phase (December 2020 - February 2021)*

By the end of the fellowship and within 3 months of completing their field research, fellows were encouraged to produce a scholarly output, which could be in the form of a journal submission, a report, or a policy brief.

Upon successful completion of the three phases, the CREEW fellows received a Certificate in Research and Education in the Ecology of War.

All research work conducted at CREEW complied with international ethical standards. As per AUB guidelines, all research projects required approval by AUB’s Institutional Review Board (IRB). CREEW fellows were required to read the IRB’s Principles and Procedures Manual during the residential courses. CREEW fellows also underwent the mandatory Collaborative Institutional Training Initiative (CITI Programme) required by IRB and AUB.

**Recruitment and Participants**

A call for application for the CREEW fellowship was promoted on all GHI’s social media platforms one week prior to the application start date. A more targeted recruitment strategy was adopted by sending out a call for applications to specific organizations, including hospitals, universities, governments, NGOs/iNGOs, and ministries, operating in areas of conflict such as: Syria, Palestine, Yemen, Iraq, and Libya.

Applicants were required to submit a letter of intent that includes a general description of the project of interest, a title for the proposed project, the population of interest, the significance of the project for the country in which it would be conducted, and an explanation of how the applicant’s professional portfolio fits the proposed project. Along with the letter of intent, a personal statement that includes the applicant’s academic and professional background, their experience in working within conflict settings, their reasons for applying to the fellowship, and a justification of how this fellowship can help in achieving the applicant’s academic and career goals were also requested. Additionally, applicants had to submit contact information of two referees who can submit recommendation letters, along with their curriculum vitae. A total of 79 applications were received.

The project coordinator reviewed incoming applications on a rolling basis and filtered out candidates based on the following eligibility criteria:

- Applicant should be a working health professional with documented experience
- Applicant should be interested in pursuing research work in conflict settings and the thematic topic
- Applicant should demonstrate a level of English proficiency consistent with the demands of the CREEW fellowship

Applications for consideration were sent to the admissions committee. Committee members individually reviewed applications and met for two sessions to decide which of the fellows were to be invited for an interview. To ensure equity during the fellow selection process, a rubric was used to rate each candidate. The rubric served as a guiding tool to filter out the most eligible candidates. The selection was then determined through reaching a consensus by the admission committee, as guided by the score generated by the tool.

In the first iteration of the programme, 7 applicants were accepted but only 5 applicants from Iraq, Palestine, Sudan, Syria, and Yemen were able to make it to Beirut as part of the first cohort.

**Table 1. Overview of Courses**

| **Course 1: Thematic Topic: Antimicrobial Resistance**  3 modules: introduction to AMR, AMR in war and conflict, AMR research applications  Online, 20 hours | The course aims to strengthen learner’s knowledge on the topic of Antimicrobial Resistance (AMR) and introduce potential knowledge gaps, which may be avenues for research proposals. |
| --- | --- |
| **Course 2: Introduction to Global Health and the Ecology of War**  3 modules: ecology of war, challenges and risks of conducting research, research ethics  In-person, 20 hours | The course provides an overview of the conceptual framework of the Ecology of War and its implications on global health research. The fellows will learn to manage and mitigate physical and digital security risks related to conducting research in conflict settings. In addition, fellows will be prompted to think through research strategies that require navigating legal and administrative landscapes in conflict settings. In this course, the fellows will be guided through the proposal writing process including ethical review and will be expected to submit their research proposals for ethical review. |
| **Course 3: Applied Methods of Quantitative Research in Conflict Settings**  6 modules: research question, literature research, research design, sampling strategies, data collection, wrap-up  In-person, 30 hours | The course introduces fellows to principles of research designs in areas of conflicts. It promotes critical thinking when developing and addressing scientific inquiries. The course initiates with the operationalization of a research question, formulating hypotheses, and finding supporting literature. The course then moves into the identification of research designs suitable for conflict areas focusing on the identification of population of interest, sampling strategies, and tools for data collection. Throughout the course difficulties specific to conflict areas will be addressed |
| **Course 4: Qualitative Research Design and Application in Conflict Settings**    4 modules: foundations of qualitative research, methods, data analysis, research proposal    In-person, 30 hours | This course explores the basis for selecting and designing qualitative research in a conflict setting. Learners will be introduced to the different qualitative research methodologies, the steps for designing a rigorous study, and the analytical journey. The course first explores the theoretical frameworks of qualitative research. Fellows will be able to demonstrate the ability to propose a qualitative research question and a suitable sampling and data collection method. The fellows will also be guided through the process of preparing a qualitative research proposal. |
| **Course 5: Quantitative Data Collection Strategies for Conflict Settings**    4 modules: data entry, data cleaning and management, descriptive data analysis, inferential data analysis    In-person, 30 hours | This course introduces basic statistical techniques applied to health sciences in conflict settings. It covers the theory and application of statistical techniques that are commonly used in clinical research in such settings. The course involves didactic teaching, hands-on computer exercises/demonstrations for collecting and analysing clinical datasets and reporting their results. The main objectives are twofold: understanding descriptive statistics, which encompass techniques for organizing and summarizing data, and understanding inferential statistics from estimation to confidence interval, and testing of hypotheses. Applications include Chi-square, Pearson correlation, Student t-test and ANOVA. |

**References**

1. The World Bank. Classification of Fragile and Conflict-Affected Situations, https://www.worldbank.org/en/topic/fragilityconflictviolence/brief/harmonized-list-of-fragile-situations (2022, accessed March 23, 2022).

2. Kohrt BA, Mistry AS, Anand N, et al. Health research in humanitarian crises: An urgent global imperative. *BMJ Global Health*; 4. Epub ahead of print November 1, 2019. DOI: 10.1136/bmjgh-2019-001870.

3. Woodward A, Sheahan K, Martineau T, et al. Health systems research in fragile and conflict affected states: a qualitative study of associated challenges. *Health Research Policy and Systems* 2017; 15: 1–12.

4. Murray CJL, King G, Lopez AD, et al. Armed conflict as a public health problem. *British Medical Journal* 2002; 324: 346–349.

5. Chaabna K, Cheema S, Abraham A, et al. The state of population health research performance in the Middle East and North Africa: a meta-research study. *Systematic Reviews*; 10. Epub ahead of print December 1, 2021. DOI: 10.1186/s13643-020-01552-x.

6. Mansour R, Naal H, Kishawi T, et al. Health research capacity building of health workers in fragile and conflict-affected settings: a scoping review of challenges, strengths, and recommendations. *Health Research Policy and Systems* 2021; 19: 1–23.

7. Clarke PK, Darcy J. *Insufficient evidence? The quality and use of evidence in humanitarian action*. London, www.alnap.org/pool/files/discussion-starter-evidence-alnap-2014.pdf (2014).

8. Blanchet K, Ramesh A, Frison S, et al. Evidence on public health interventions in humanitarian crises. *The Lancet* 2017; 390: 2287–2296.

9. Naal H, el Koussa M, el Hamouch M, et al. A systematic review of global health capacity building initiatives in low-to middle-income countries in the Middle East and North Africa region. *Globalization and Health* 2020; 16: 1–16.

10. Naal H, el Koussa M, el Hamouch M, et al. Evaluation of global health capacity building initiatives in low-and middle-income countries: A systematic review. *Journal of Global Health* 2020; 10: 020412.

11. O’Neill J. *Tackling drug-resistant infections globally: final report and recommendations.* London, 2016.

12. Murray CJ, Ikuta KS, Sharara F, et al. Global burden of bacterial antimicrobial resistance in 2019: a systematic analysis. *The Lancet*. Epub ahead of print January 2022. DOI: 10.1016/S0140-6736(21)02724-0.

13. Nawfal Dagher T, Al-Bayssari C, Diene SM, et al. Bacterial infection during wars, conflicts and post-natural disasters in Asia and the Middle East: a narrative review. *Expert Review of Anti-Infective Therapy* 2020; 18: 511–529.

14. O’Shea MK. Acinetobacter in modern warfare. *International Journal of Antimicrobial Agents* 2012; 39: 363–375.

15. Murray CK. Epidemiology of infections associated with combat-related injuries in Iraq and Afghanistan. *The Journal of Trauma*; 64. Epub ahead of print March 2008. DOI: 10.1097/TA.0B013E318163C3F5.

16. Devi S. AMR in the Middle East: “a perfect storm.” *The Lancet* 2019; 394: 1311–1312.
